# Receptive Field Inference with Localized Priors

**DOI:** 10.1371/journal.pcbi.1002219

**Published:** 2011-10-27

**Authors:** Mijung Park, Jonathan W. Pillow

**Affiliations:** 1 Department of Electrical and Computer Engineering, The University of Texas at Austin, Austin, Texas, United States of America; 2 Center for Perceptual Systems, Department of Psychology and Section of Neurobiology, The University of Texas at Austin, Austin, Texas, United States of America; Indiana University, United States of America

## Abstract

The linear receptive field describes a mapping from sensory stimuli to a one-dimensional variable governing a neuron's spike response. However, traditional receptive field estimators such as the spike-triggered average converge slowly and often require large amounts of data. Bayesian methods seek to overcome this problem by biasing estimates towards solutions that are more likely *a priori*, typically those with small, smooth, or sparse coefficients. Here we introduce a novel Bayesian receptive field estimator designed to incorporate *locality*, a powerful form of prior information about receptive field structure. The key to our approach is a hierarchical receptive field model that flexibly adapts to localized structure in both spacetime and spatiotemporal frequency, using an inference method known as empirical Bayes. We refer to our method as *automatic locality determination* (ALD), and show that it can accurately recover various types of smooth, sparse, and localized receptive fields. We apply ALD to neural data from retinal ganglion cells and V1 simple cells, and find it achieves error rates several times lower than standard estimators. Thus, estimates of comparable accuracy can be achieved with substantially less data. Finally, we introduce a computationally efficient Markov Chain Monte Carlo (MCMC) algorithm for fully Bayesian inference under the ALD prior, yielding accurate Bayesian confidence intervals for small or noisy datasets.

## Author Summary

A central problem in systems neuroscience is to understand how sensory neurons convert environmental stimuli into spike trains. The receptive field (RF) provides a simple model for the first stage in this encoding process: it is a linear filter that describes how the neuron integrates the stimulus over time and space. A neuron's RF can be estimated using responses to white noise or naturalistic stimuli, but traditional estimators such as the spike-triggered average tend to be noisy and require large amounts of data to converge. Here, we introduce a novel estimator that can accurately determine RFs with far less data. The key insight is that RFs tend to be localized in spacetime and spatiotemporal frequency. We introduce a family of prior distributions that flexibly incorporate these tendencies, using an approach known as empirical Bayes. These methods will allow experimentalists to characterize RFs more accurately and more rapidly, freeing more time for other experiments. We argue that locality, which is a structured form of sparsity, may play an important role in a wide variety of biological inference problems.

## Introduction

A fundamental problem in systems neuroscience is to determine how sensory stimuli are functionally related to a neuron's response. A popular mathematical description of this encoding relationship is the “cascade” model, which consists of a linear filter followed by a noisy nonlinear spiking process. The linear stage in this model is commonly identified as the neuron's *spatiotemporal receptive field*, which we will refer to simply as the receptive field (RF) or “filter”. The RF describes how a neuron sums up its inputs across space and time. It can also be conceived as the spatiotemporal stimulus pattern that optimally drives the neuron to spike. A large body of literature in sensory neuroscience has addressed the problem of estimating a neuron's RF from its responses to a rapidly fluctuating stimulus, a problem known generally as “neural characterization” [1]–[17].

Here we focus on a highly simplified encoding model that describes neural responses in terms of a linear filter and additive Gaussian noise [5], [11], [18]. Although this model gives an imperfect description of real neural responses, the RF estimators that arise from it (such as the spike-triggered average) are consistent under a much larger class of models [7], [19], [20]. The maximum likelihood filter estimate under the linear-Gaussian model is the whitened spike-triggered average (STA), also known as *linear regression*, *reverse correlation*, or the *first-order Weiner kernel* [1]–[3]. The STA has an extensive history in neuroscience and has been used to characterize RFs in a wide variety of areas, including retina [4], [7], [13], [21], [22], lateral geniculate nucleus [23], [24], primary visual cortex [5], [25], and peripheral as well as central auditory brain areas [8], [9], [11], [26]–[28].

The STA is often high-dimensional (containing tens to hundreds of parameters) and generally requires large amounts of data to converge. With naturalistic stimuli, the whitened STA is often corrupted by high-frequency noise because natural scenes contain little power at high frequencies. A common solution is to regularize the filter estimate by penalizing unlikely parameter settings, generally by biasing parameters towards zero (also known as “shrinkage”). Statisticians have long known that biased estimators can achieve substantially lower error rates in high-dimensional inference problems [29], [30], and Bayesian methods formalize such biases in terms of a prior distribution over the parameter space. In neuroscience applications, priors for sparse (having many zeros) or smooth (having small pairwise differences) filter coefficients have been used to obtain substantially more accurate RF estimates [9], [11], [12], [15], [31].

However, neural receptive fields are more than simply sparse or smooth. They are *localized* in both spacetime and spatiotemporal frequency. This is a structured form of sparsity: RFs contain many zeros, but these zeros are not uniformly distributed across the filter. Rather, the zeros tend to occur outside some region of spacetime and, in the Fourier domain, outside some region of spatiotemporal frequency. Although this property of receptive fields is well-known [32], [33], it has not to our knowledge been previously exploited for receptive field inference. Here we introduce a family of priors that can flexibly encode locality. Our approach is to first estimate a localized prior from the data, and then find the maximum a posteriori (MAP) filter estimate under this prior. This general approach is known in statistics as parametric empirical Bayes [34], [35]. Our method is directly inspired by previous empirical Bayes estimators designed to incorporate sparsity [36] and smoothness [11]. We show that locality can be an even more powerful source of prior information about neural receptive fields, and introduce a method for simultaneously inferring locality in two different bases, yielding filter estimates that are both sparse (local in a spacetime basis) and smooth (local in a Fourier basis).

## Results

The results section is organized as follows. First, we will describe the linear-Gaussian encoding model and the empirical Bayes framework for receptive field estimation. Second, we will review several previous empirical Bayes RF estimators, to which we will compare our method. Third, we will derive three new receptive field estimators that we collectively refer to as *automatic locality determination* (ALD). We will apply ALD to simulated data and to neural data recorded in primate V1 and primate retina. Finally, we will describe an extension from empirical Bayes to “fully Bayesian” inference under the ALD prior.

### Model-based receptive field estimation

A typical neural characterization experiment involves rapidly presenting stimuli from some statistical ensemble and recording the neuron's response in discrete time bins. Let 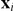 denote the (vector) stimulus and 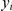 the neuron's (scalar) spike response at time bin 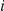. Here, 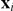 is a vector of spacetime stimulus intensities over some preceding time window that affects the spike response at time bin 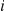.

**Figure 1:**
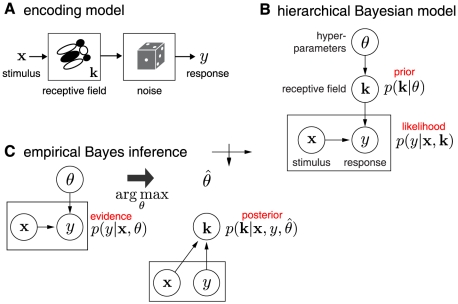
Neural encoding model and empirical Bayes receptive field inference. (**A**) Linear Gaussian encoding model: the stimulus 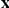 is projected on the receptive field 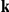 and Gaussian noise is added to produce the neural response 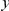. (**B**) Graphical model for a hierarchical Bayesian receptive field model. The hyperparameters 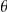 specify a prior over the receptive field 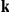, which together with stimulus 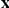 determines the conditional probability of neural response 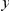. Circles indicate variables, arrows indicate conditional dependence, and the square denotes a pair of variables (stimulus 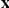 and response 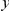) that are observed many times. (**C**) Empirical Bayes involves a two-stage inference procedure: first, maximize the evidence 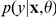 for 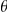 (*left*), which can be computed by integrating out 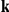 from the generative model in (B); second, maximize the posterior over 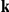 given the data and estimated hyperparameters 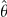 (*right*). See text for details.

We will model the neuron's response as a linear function of the stimulus plus Gaussian noise:
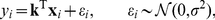
(1)where 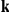 denotes the neuron's receptive field and 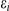 is a sample of zero-mean, independent Gaussian noise with variance 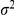. This model is the simplest type of cascade encoding model (depicted in Fig. 1 A), and plays an important role in the theory of neural encoding and decoding [5], [11], [17], [28], [37], [38]. For a complete dataset with 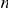 stimulus-response pairs, likelihood is given by
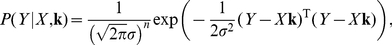
(2)where 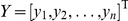 is a column vector of neural responses and 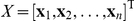 is the stimulus design matrix, with 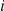
'th row equal to 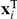. The maximum likelihood (ML) receptive field estimate is:
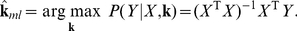
(3)This estimate, also known as the whitened spike-triggered average, and is proportional to the ordinary spike-triggered average if the stimulus ensemble is uncorrelated, meaning 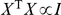.

A major drawback of the maximum likelihood estimator is that it typically requires large amounts of data to converge, especially when 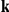 is high-dimensional. This problem is exacerbated for correlated or naturalistic stimulus ensembles, because the high-frequency components of 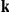 are not well constrained by the data. In the Bayesian framework, regularization is formalized in terms of a prior distribution 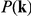, which tells us that we should bias our estimate of 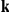 toward regions of parameter space that are more probable *a priori*. The posterior distribution, which captures the combination of likelihood and prior information, is given by Bayes' rule:
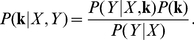
(4)The most probable filter given the data and prior is known as the *maximum a posteriori* (MAP) estimator:
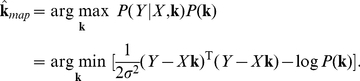
(5)The log prior behaves as a “penalty” on the solution to an ordinary least-squares problem, forcing a tradeoff between minimizing the sum of squared prediction errors and maximizing 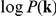.

Biased estimators can achieve substantial improvements over the maximum likelihood, particularly for high-dimensional problems, without giving up desirable features such as consistency (i.e., converging to the correct value in the limit of infinite data). However, the important question arises: how should one select a prior distribution? (Choosing the *wrong* prior can certainly lead to a worse estimate!)

One common method is to set the prior (or “penalty”) by cross-validation. This involves dividing the data into a “training” and “test” set, and selecting the prior for which 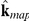 (estimated on the training set) achieves maximal performance on the test set. However, this approach is computationally expensive and may be intractable for a prior with multiple hyperparameters. Empirical Bayes is an alternative method for prior selection that does not require separate training and test data.

### Empirical Bayes

Empirical Bayes can be viewed as a maximum-likelihood procedure for estimating the prior distribution from data. It is also known in the literature as *evidence optimization*, *Type II maximum likelihood*, and *maximum marginal likelihood* [11], [34], [39]–[41]. The basic idea is that we can compute the probability of the data given a set of hyperparameters governing the prior by “integrating out” the model parameters. This probability is really just a likelihood function for the hyperparameters, so maximizing it results in a maximum-likelihood estimate for the hyperparameters. (Technically, this is *parametric empirical Bayes*, since we will assume a particular parametric form for the prior; see [34], [35], [42] for a more general discussion).

Let 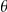 denote a set of hyperparameters controlling the prior distribution over 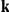, which we will henceforth denote 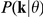. The posterior distribution over the RF (eq.4) can now be written:
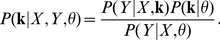
(6)The denominator in this expression is known as the *evidence* or *marginal likelihood*. (Note that we ignored this denominator when finding the MAP estimate (eq.5), since it does not involve 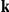). The evidence is the probability of the responses 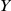 given the stimuli 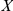 and the hyperparameters 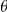, which we can compute by integrating the numerator (eq.6) with respect to 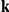:
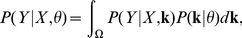
(7)where 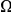 is the parameter space for 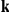. Maximizing the evidence for 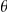 therefore amounts to a maximum likelihood estimate of the hyperparameters. The MAP estimate for 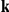 under this prior is an empirical Bayes estimate, since the prior is learned “empirically” from the data.

Empirical Bayes can therefore be described as a two-stage procedure: **(1)** Maximize the evidence to obtain 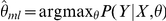; **(2)** Find the MAP estimate for 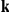 under the prior 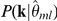. Fig. 1 shows a diagram for this hierarchical receptive field model the steps for empirical Bayesian inference.

### Zero-mean Gaussian priors

Following earlier work [11], [36], [43], [44], we will take the prior distribution to be a Gaussian centered at zero:
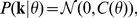
(8)where 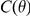 is a covariance matrix that depends on hyperparameters 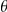 in some yet-to-be-specified manner. This Gaussian prior together with a Gaussian likelihood (eq.2) ensures the posterior is also Gaussian:
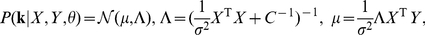
(9)where 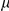 and 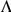 are the posterior mean and covariance. The MAP filter estimate 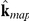 is simply the posterior mean 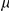, since the mean and maximum of a Gaussian are the same. Moreover, the evidence (eq.7) can be computed in closed form, since it is the integral of a product of two Gaussians. This allows for rapid optimization of 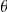. We will in practice maximize the log-evidence, given by:
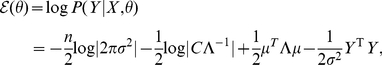
(10)where 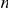 is the number of samples (rows) in 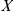 and 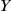. All that remains is to specify the prior covariance 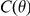, which we will explore in detail below.

Before continuing, we wish to distinguish two distinct notions of “dimensionality” for a receptive field. First, dimensionality may refer to the number of parameters or coefficients in 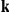. We will refer to this as the *parameter dimensionality* of the filter, denoted 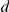. Second, dimensionality may refer to the dimensionality of the coordinate space in which the filter is defined. In this sense, a filter with 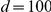 elements arranged as a 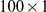 vector is 1-dimensional (e.g., a temporal filter), while a filter with the same number of elements arranged in a 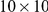 matrix is 2-dimensional (e.g., an image filter). We will refer to this as the *coordinate dimensionality* of the filter, denoted 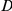.

### Previous methods

**Figure 2:**
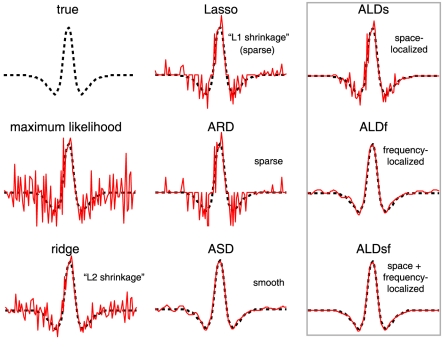
Comparison of estimators for 1D simulated example. A 1D difference-of-Gaussians receptive field with 100 elements was stimulated with 2000 samples of correlated (1/F) Gaussian noise. **Left column:** True filter (top), maximum likelihood (linear regression) estimate (middle), and empirical Bayes ridge regression (L2-penalized) estimate (bottom). **Middle:** Lasso (L1-penalized) estimate (top) and ARD (middle) produce sparse estimates but fail to capture smoothness. The ASD estimate (bottom) captures smoothness, but exhibits spurious oscillations in the tails. **Right column**: Three variants of automatic locality determination (ALD): Spacetime localization (ALDs, top), which identifies a spatial region in which the filter coefficients are large; frequency localization (ALDf, middle), which identifies a local region of the frequency domain in which Fourier coefficients are large, leading to a smooth estimate that closely resembles ASD; and joint localization in spacetime and frequency (ALDsf, bottom), which simultaneously identifies a local region in spacetime and frequency, yielding an estimate that is both smooth and sparse.

We will examine three empirical Bayes RF estimators from the literature: ridge regression [45], Automatic Relevance Determination (ARD) [36], [43], [44], and Automatic Smoothness Determination (ASD) [11]. Fig. 2 provides an illustrative comparison of these methods, using a simulated example consisting with a 100-element vector filter (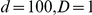), stimulated with correlated (“1/F”) Gaussian noise stimuli. The true filter was a difference of two Gaussians, and the maximum likelihood estimate (middle left) is badly corrupted by high frequency noise.

First, *ridge regression* assumes a prior with covariance matrix proportional to the identity matrix: 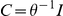. This treats the filter coefficients as drawn *i.i.d.* from a zero-mean Gaussian prior with precision (“inverse variance”) 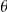. Ridge regression is penalized least-squares estimate with a penalty (eq.5) on the squared 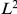 norm of the filter, given by 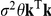. This penalty shrinks the coefficients of 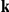 towards zero. Larger 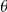 yields smaller filter coefficients, and in the limit of infinite 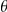, the MAP estimate shrinks to all-zeros. Set correctly, the ridge prior can provide substantial improvement over maximum likelihood, especially when the stimulus autocovariance is ill-conditioned, as it is for naturalistic stimuli (see Fig. 2). Ridge regression is perhaps the most popular and well-known regularization method. Although it is not usually employed in an empirical Bayes framework, it is straightforward (and fast) to maximize the evidence for the ridge parameter 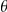 using a fixed-point rule [36], [45]. (See Methods).

Second, *Automatic Relevance Determination* (ARD) [36] assumes a diagonal prior covariance matrix with a distinct hyperparameter 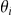 for each element of the diagonal. This resembles the ridge prior covariance except that the prior variance of each filter coefficient is set independently. The prior covariance matrix can be written 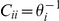, where 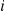 ranges over the number of elements in 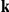. It would be intractable to use cross-validation to estimate all the elements in 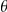 (a 100-element vector in Fig. 2), so empirical Bayes plays a critical role for inference. In practice, evidence maximization drives many of the prior variances to zero, making the posterior a delta function at zero for those coefficients. The MAP estimate for these coefficients is therefore zero, making the ARD estimate sparse. The ARD estimate can be computed rapidly using fixed-point methods, expectation-maximization, or variational methods [43], [44], [46]–[49]. Fig. 2 (middle column) shows the ARD and the *lasso* estimate [50], the latter of which is the MAP estimate under an exponential (or 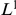) prior. We set the lasso parameter here by cross-validation. Both estimates are sparse. The ARD estimate is actually sparser and less biased towards zero for large coefficients, but both fail to provide a close match to the smooth filter used in this example.

Third, *Automatic Smoothness Determination* (ASD) [11] assumes a non-diagonal prior covariance, given by a Gaussian kernel [51], which is parametrized so that the correlation between filter coefficients falls off as a function of their separation distance. The rationale here is that RFs are smooth in both space and time, so nearby coefficients should be highly correlated, while more distant ones should be more nearly independent. For a 1D filter, the ASD prior covariance takes the form of a “fuzzy ridge”, with Gaussian decay on either side of the diagonal. The 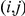
'th element is given by 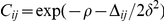, where 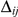 is the squared distance between the filter coefficients 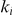 and 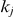 in pixel space, and the hyperparameters 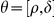 control the scale (analogous to the ridge parameter) and smoothness (the width of the fuzzy ridge), respectively. For filters with higher coordinate dimension (e.g., a 2D spatial filter), the hyperparameters include additional hyperparameters to control smoothness in each direction. Optimization of 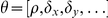 can be achieved by gradient ascent of the log-evidence (see Methods). For our simulated example (Fig. 2, bottom middle), the ASD estimate is indeed smooth due to the correlations in the inferred prior.

Note that for smooth RFs, the ASD prior covariance matrix becomes ill-conditioned, as some of its eigenvalues are very close to zero. This implies that the ASD estimate is sparse, but (unlike ARD) it is not sparse in the pixel basis. Rather, the ASD estimate is sparse in a basis that depends on the hyperparameters (since the eigenvectors of the ASD prior covariance vary with the hyperparameters). The small-eigenvalue eigenvectors tend to have high-frequency oscillations, meaning that the ASD estimate is sparse in a Fourier-like basis, with the prior variance of high-frequency modes set near to zero. In our view, ASD is the current state-of-the-art method for linear filter estimation and indeed (as shown in Fig. 2) it performs far better than previous methods for realistic neural RFs.

### Automatic Locality Determination (ALD)

The motivation for our approach is the observation that neural receptive fields tend to be localized in space, time, and spatiotemporal frequency (i.e., Fourier space). Neurons in the visual pathway, for example, tend to integrate light only within some restricted region of visual space and some finite window of time, and respond only to some finite range of spatiotemporal frequencies [25], [32], [52], [53]. This is tantamount to a structured form of sparsity: large groups of coefficients (e.g., those outside some spacetime region) that fall to zero in a dependent manner. Here we describe three prior distributions for exploiting this structure. We refer to these methods collectively as *automatic locality determination (ALD)*.

#### Locality in spacetime (ALDs)

First we formulate a prior covariance matrix 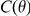 that can capture the tendency for RFs to have a limited extent in space and time. We can achieve this with a diagonal covariance matrix, but instead of using a constant diagonal (as in ridge regression) or a vector of hyperparameters along the diagonal (as in ARD), we use a functional form for the diagonal that allows the prior variance to be large for coefficients within some region, and small (decaying to zero) for coefficients outside that region.

We parametrize the local region with a Gaussian form, so that prior variance of each filter coefficient is determined by its Mahalanobis distance (in coordinate space) from some mean location 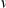 under a symmetric positive semi-definite matrix 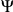. The diagonal prior covariance matrix is given by:
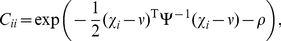
(11)where 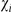 is the spacetime location (i.e., filter coordinates) of the 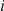
'th filter coefficient 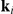, 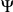 is a covariance matrix determining the shape and extent of the local region, and 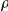 sets the overall scale of the prior variance (as in ASD). We refer to this method as ALDs, for automatic locality determination in *spacetime coordinates*. The hyperparameters governing the ALDs prior are 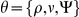, which can specify an arbitrary elliptical region of coordinate space where prior variance is large.

**Figure 3:**
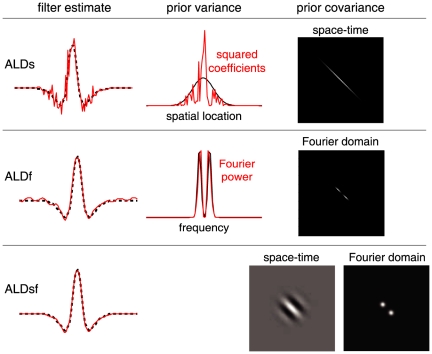
Estimated filters and prior covariances for ALD methods. (Same example filter as shown in Fig. 2). Left column shows the true filter (dotted black) and ALD estimates (red) replotted from the right-most column of Fig. 2. **Top:** Space-localized estimate. The estimated prior variance (black trace, middle) is a Gaussian form that controls the falloff in amplitude of filter coefficients (red) as a function of position. The prior covariance (right) is a diagonal matrix with this Gaussian along the diagonal. The prior is thus independent with location-dependent variance. **Middle:** Frequency-localized estimate. A Gaussian form (reflected around the origin due to symmetries of the Fourier transform) specifies the prior variance as a function of frequency (black trace, middle). The Fourier power of the filter estimate (red) drops quickly to zero outside the estimated region. The prior covariance matrix (right) is diagonal in the Fourier domain, meaning the Fourier coefficients are independent with frequency-dependent variance. **Bottom:** Space and frequency localized estimate. The estimated prior covariance matrix is not diagonal in spacetime or frequency, but takes the form of a “sandwich matrix” that combines the prior covariances from ALDs and ALDf (see text). The resulting prior covariance matrix can be visualized in either the spacetime domain (left) or the Fourier domain (right). It is localized (has a local region of large prior variance) in both coordinate frames, but has strong dependencies (off-diagonal elements), particularly across space.

Fig. 2 shows the ALDs estimate for the 1D example discussed above. As expected, the RF coefficients are large within a central region, and decay to zero outside it. Fig. 3 (top row) shows the prior variance underlying this estimate (i.e., the diagonal of the prior covariance matrix 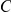) at the maximum-evidence 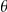. The method can be extended to filters of higher coordinate dimensionality 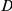. In this case, with 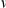 is a 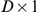 vector and 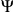 is a 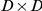 symmetric, positive definite matrix specified by 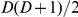 parameters.

Computationally, ALDs is faster than ASD because, although its parametrization is similar, the prior covariance matrix is diagonal. As the localized region described by the hypearparemeters becomes smaller, the prior variance of outer filter pixels falls arbitrarily close to zero, and we can prune these coefficients (as in ARD) because the prior effectively pins them to zero. This reduces the dimensionality of 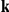, making it sparse in pixel space, and making evaluation of the log-evidence (eq.10) faster. The key difference from ARD, however, is that pruning does not take place independently for each coefficient, but occurs systematically as a function of distance from 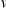, the center of some spatiotemporal region.

Note that the ALDs estimator does not assume any functional form for the filter itself. Rather, it seeks to determine (via evidence optimization) only whether there is some elliptical region beyond which the filter coefficients fall to zero. If an RF is *not* localized, the evidence will be maximal when the width of the region specified by 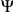 becomes much larger than the area covered by the RF coefficients. In this limit, the diagonal of the prior covariance will be nearly constant, where the ALDs prior is equivalent to the ridge regression prior.

Although ALDs correctly identifies spacetime locality in simulated examples, the estimates it provides are not smooth. The use of a diagonal prior covariance 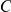 means that the filter coefficients are independent *a priori* given 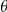. We can address this shortcoming by considering a different basis for the RF coefficients.

#### Locality in frequency (ALDf)

Neural receptive fields are localized in spatiotemporal frequency as well as in spacetime, which is apparent from their Fourier power spectra [53]. That is, a neuron typically responds to sine waves over some limited range of spatiotemporal frequencies, and is insensitive beyond this range. We can design a prior covariance matrix to capture this structure by employing the ALDs prior in the Fourier domain. We refer to this as the ALDf, for automatic locality determination in *frequency coordinates*.

We can define a Gaussian prior over the Fourier-transformed RF coefficients 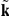 using a diagonal covariance matrix 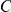 with diagonal:
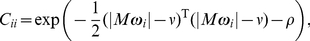
(12)where 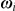 denotes the frequency coordinates for the 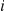
'th coefficient of 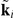, 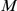 is a symmetric matrix, and 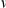 describes the mean of (symmetric) elliptical regions in Fourier space. The absolute value ensures reflection symmetry through the origin, a property of the Fourier transform of any real signal, while allowing localized Fourier energy to exhibit orientations in spacetime and to extend over different frequency ranges for different coordinate dimensions. The hyperparameters are 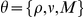. (See Methods for details).

The ALDf estimate can be computed efficiently by taking the discrete Fourier transform of stimuli 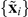, maximizing evidence for 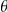 under the diagonal ALDf prior (eq.10), computing 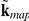 in the Fourier domain (eq.9), and then taking the inverse Fourier transform to obtain the spacetime filter 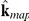. (See Methods). Note that a filter in 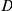 coordinate dimensions requires the 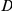-dimensional Fourier transform. Fig. 2 shows the ALDf estimate for the simulated 1D example, and Fig. 3 (second row) shows the diagonal of the estimated prior covariance and Fourier spectrum of the ALDf estimate, which exhibits modes at 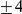 Hz. Note that this filter is more sparse in the Fourier domain than the space domain, and that the ALDf estimate exhibits correspondingly smaller error than the ALDs. Thus, locality in frequency is more useful than locality in spacetime for smooth RFs.

Although the ASD and ALDf estimates look similar for this 1D example, the latter achieves slightly lower error due to the fact that it also suppresses low frequencies (e.g., the DC component), which are also small for this filter. The ASD prior, in contrast, always assigns highest prior variance to the lowest frequency Fourier components. (This can be seen by inspecting the ASD prior covariance matrix in the Fourier basis). ALDf can be expected to outperform ASD whenever the Fourier spectrum is not a monotonically decreasing function of frequency; however, for realistic examples we considered, the two perform very similarly. The main limitation of both methods is a failure to account for locality in spacetime, which is evident in the ripples present in the tails of both estimates (Fig. 2).

#### Locality in spacetime and frequency (ALDsf)

The two methods described above exploit locality by estimating a diagonal prior covariance matrix in either a spacetime basis (ALDs) or a Fourier basis (ALDf). However, neural receptive fields generally exhibit both kinds of locality at once. One would therefore like to design a prior that simultaneously captures both forms of locality. We can accomplish this by forming a “sandwich” matrix out of the two prior covariance matrices defined above. We define the prior covariance to be:
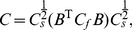
(13)where 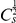 is the square root of the diagonal ALDs prior covariance (eq.11), 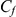 is the diagonal ALDf prior covariance matrix (eq.12), and 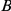 is an orthogonal basis matrix for the 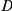-dimensional discrete Fourier transform. (That is, 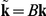, 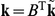, and 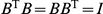.) This formulation effectively imposes the two forms of locality in series: first, the spacetime prior covariance (outer matrix); then Fourier transform and the frequency domain covariance (inner matrix). Although there are other combination schemes are possible (see Discussion), we found this one to give the best performance on simulated data. We call the resulting estimate ALDsf, for automatic locality determination in *spacetime and frequency*.

The hyperparameters for ALDsf are union of the ALDs and ALDf hyperparameters, 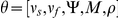, where subscripts 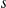 and 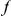 indicate parameters for the spatial and frequency domain matrices 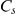 and 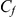, respectively. We perform evidence optimization over this full set of hyperparameters, although it is helpful to initialize with the values estimated for each of the two above methods individually to avoid sub-optimal local maxima. Fig. 2 (bottom right) shows the ALDsf estimate for our 1D example, which is nearly indistinguishable from the true filter. Fig. 3 shows the estimated prior covariance matrix 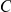, represented in both pixel and Fourier bases. As expected, the prior covariance exhibits locality in both coordinates (bases), but is no longer diagonal in either. This indicates that the resulting prior covariance imposes dependencies between neighboring coefficients in both 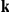 and its Fourier transform 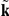.

One useful feature of ALDsf is that it defaults to ALDf if the filter is not localized in space, to ALDs if not localized in frequency, or to ridge regression if not localized in either basis. When the filter is not localized, the evidence will favor regions that are sufficiently broad (i.e., sufficiently large 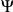 and 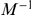) that the matrices 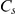 or 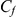 (or both) will approximate the identity matrix, eliminating the prior preference for locality in the corresponding basis. When both 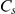 and 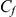 are the identity matrix, the resulting covariance matrix 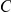 corresponds to the ridge regression prior.

### Application to simulated data

**Figure 4:**
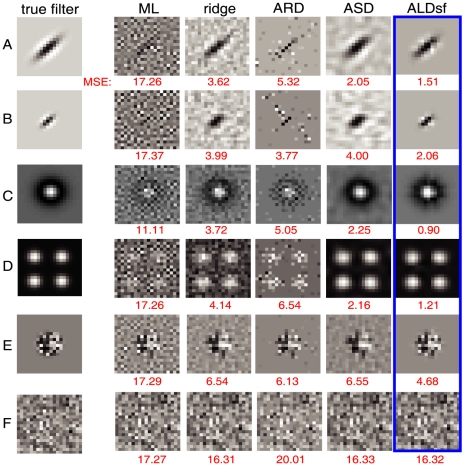
Menagerie of simulated examples. Noisy responses to 1600 random 1/F Gaussian stimuli were simulated and used for training. The leftmost column shows the true filter (a 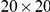 pixel image), while subsequent columns show various estimates. The mean squared error of each estimate is indicated below in red. Filters shown include: (**A**) Oriented Gabor filter, typical of a V1 simple cell; (**B**) Smaller Gabor filter; (**C**) center-surround “difference-of-Gaussians” filter, typical of retinal ganglion cells; **D**) grid cell with multiple non-zero regions (localized in the Fourier domain but not in space); (**E**) circularly windowed Gaussian white noise (localized in space but not in frequency); (**F**) full field Gaussian noise (not localized in space or frequency). ALDsf performs at or near the optimum for all examples we examined.

To compare performance with previous receptive field estimators, we began with simulated data. We generated six different 2D spatial receptive fields with varying degrees of locality in space and frequency. Each filter consisted of a 2D array of 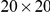 pixels, making for a parameter space of 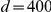 dimensions. Noisy responses were simulated using 1600 samples of 1/F correlated Gaussian noise according to (eq.1). Results are shown in Fig. 4.

Each row of Fig. 4 shows one of the six filters, and the estimates provided by maximum likelihood (ML), ridge regression, ARD, ASD, and (highlighted in blue) ALDsf. The numbers in red below each estimate indicate the mean squared error between the true filter and the estimate. (We did not show ALDs or ALDf because ALDsf always performed best of the three new methods). The simulated examples included: (A) a large Gabor filter; (B) a small Gabor filter; (C) a retina-like center-surround RF; (D) a grid cell RF with several non-zero regions; (E) circularly windowed Gaussian white noise; and (F) a pure Gaussian white noise filter. The grid cell filter did not exhibit strong locality in space, while the windowed white noise did not exhibit locality in frequency, and the pure white noise filter did not exhibit locality in either space nor frequency. Nevertheless, the ALDsf estimate had the smallest error by a substantial margin for all examples except the white noise filter. For the white noise filter, the ridge prior (i.i.d. zero-mean Gaussian) was in fact the “correct” prior. For this example, the ASD and ALDsf estimates were not distinguishable from the ridge regression estimate, consistent with the expectation that both should default to the ridge prior when the evidence did not favor smoothness (ASD) nor locality (ALDsf).

**Figure 5:**
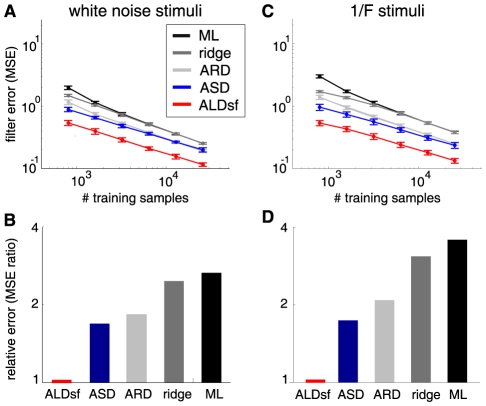
Comparison of error rates on simulated data. Responses of a 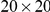 pixel Gabor filter (shown in Fig. 4 A) were simulated using white noise stimuli (left) or “naturalistic” 1/F Gaussian stimuli (right). (**A**): Filter error using white noise stimuli, for varying amounts of training data (See Methods). (**B**) Average filter error under each method. (**C–D**) Analogous to A–B, but for 1/F stimuli. For both kinds of stimuli, ALDsf achieved error rates almost 2 times smaller than ASD, the next best method. By examining horizontal slices through panels (A) and (C), it is apparent that traditional methods (ML and ridge regression) required four times more data on white noise stimuli, and twenty to thirty times more data on 1/F stimuli, to achieve the same error rate as ALDsf.

We examined the convergence properties of the various estimators as a function of the amount of data collected. We simulated responses from the first filter in Fig. 4A according to (eq.1), using two kinds of stimuli: Gaussian white noise, and 1/F correlated Gaussian noise, which more closely resembles natural stimuli. The results (Fig. 5) show that the ALDsf estimate achieved the smallest error for both kinds of stimuli, regardless of the number of training samples. The upper plots in Fig. 5 show that for white noise stimuli, traditional estimators (ML and ridge regression) needed more than four times more data than ALDsf to achieve the same error rate. For naturalistic stimuli, traditional estimators needed twenty to thirty times more data. The bottom row of plots shows the ratio of the average mean-squared error (MSE) for each estimate to the average MSE for the ALDsf estimate, showing that the next best method (ASD) exhibits errors nearly 1.8 times larger than ALDsf.

### Application to neural data

Next, we compared the various estimators using neural data recorded from simple cells in primate V1 [53]. The stimuli consisted of 16 “flickering bars” aligned with each cell's preferred orientation. We took the receptive field to have a length of 16 time bins, resulting in a 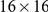 filter with two coordinate dimensions (space
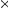
time), resulting in a 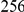-dimensional parameter space. Because the “true” filter was not known, we quantified performance using relative cross-validation error, defined as the prediction error on an 8-minute test set (See Methods). We varied the amount of data used for training, and performed 100 repetitions with randomly selected subsets of the full training data to obtain accurate estimates for each size training set.

**Figure 6:**
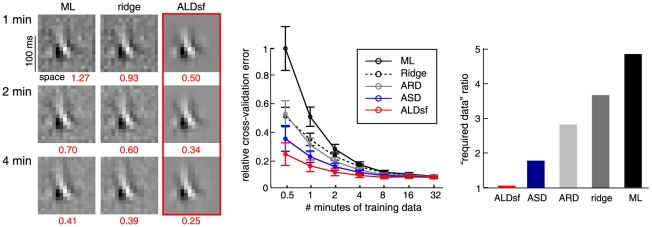
Receptive field estimates for V1 simple cells. (Data from [53]). **Left**: Filter estimates obtained by ML, ridge regression, and ALDsf, for three different amounts of training data (1, 2, and 4 min). Numbers in red beneath each filter indicate relative cross-validation error. **Middle**: Relative cross validation error for each method, averaged across 16 neurons. ALDsf achieved the lowest average error, for all amounts of training data. **Right**: Number of times more training data required by each method to obtain the same error level of as ALDsf with 30s of training data. On average, the ML estimator required 5 times more training data, while ASD required 1.7 times more training data to match the performance of ALDsf.

Fig. 6 (left) shows ML, ridge regression and ALDsf estimates for an example cell with a 1, 2 or 4 minutes of training data. Numbers in red indicate the average cross-validation error of each estimate. Note that with only 1 minute of data, ALDsf performed nearly as well as ML and ridge regression with 4 minutes of data. The middle panel shows a summary of cross-validation error for each of the five empirical Bayes estimators discussed previously, as a function of the amount of training data. ALDsf once again achieved substantially lower error than other methods. The right panel shows how many times more data were required to achieve the same level of cross-validation error as ALDsf. On average, ALDsf required 1.7 times less data than the next best method (ASD) and five times less data than maximum likelihood.

**Figure 7:**
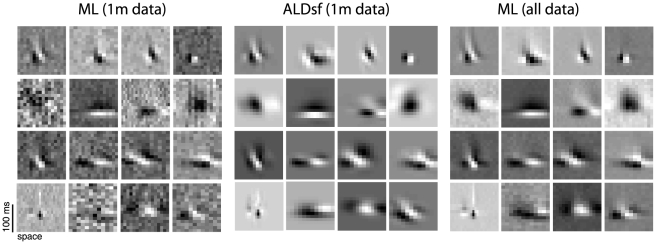
Receptive field estimates for the full set of sixteen V1 simple cells analyzed. (Data from [53]). **Left**: ML filter estimates from 1 minute of training data. **Middle**: ALDsf estimates from 1 minute of training data. **Right**: ML estimates from all data (an average of approximately 40 minutes of data per cell). Note the heterogeneity across cells, and that ALDsf captures the qualitative RF structure even when the 1-minute ML estimate is nearly indistinguishable from noise.

Fig. 7 shows the ML and ALDsf estimates for all 16 V1 simple cells in the population obtained with 1 minute of training data, as well as the ML estimate obtained using all the data available for each cell (40 minutes of data, on average). Note that for ALDsf recovers the qualitative structure of these RFs even when the underlying RF structure is barely discernible in the 1-minute ML estimate. Also note that the population exhibits substantial variability in RF shape, with many neurons whose RFs would not be well described by a fixed parametric form such as a Gabor filter.

**Figure 8:**
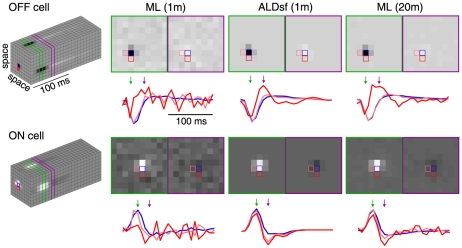
Comparison of 3D receptive field estimates for retinal data. (Data from Chichilnisky lab, [55]). **Top row**: Maximum likelihood and ALDsf estimates for an OFF retinal ganglion cell (RGC) receptive field, stimulated using 1 minute of binary spatiotemporal white noise. Left column shows a schematic of the 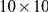 pixel 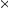
25 time bin receptive field, containing 2500 total coefficients. Each time bin was 8.33 ms, corresponding to a frame rate of 120 Hz. Colored lines indicate specific pixels whose timecourses shown at right, and spatial time-slices, depicted as images at right (taken at the 4th and 8th time bins, indicated by green and purple arrows, respectively). The ML and ALDsf estimates with 1 minute of training data are shown alongside the ML estimate computed from 20 minutes of data. Pixel time-courses were rescaled to be unit vectors, so that differences in temporal profiles (i.e., spacetime non-separability of filter) can be observed. **Bottom row**: Similar plots for an ON RGC, with spatial profiles shown for the 5th and 8th time bins. In both cases, the ALDsf accurately recovered the shape and timecourse of the RF, while the ML estimate was often indistinguishable from noise. We examined RF estimates from 3 ON and 3 OFF cells, and found that, with 1 minute of training data, the average mean-squared-error between each estimate and a reference estimate (the ML estimate computed with 20 minutes of data) was 18 times larger for ML and 6.6 times larger for ridge regression than for ALDsf.

We examined a second dataset of retinal ganglion cells (RGCs) in primate retina, which stimulated with 2D spatiotemporal white noise (“binary flicker”) [54], [55]. The RFs considered had 3 coordinate dimensions (space
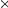
space
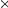
time), and a 2500-dimensional parameter space (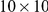 pixels in space
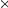
25 8.33 ms-bins in time). Fig. 8 shows the spatial (2D) and the temporal (1D) slices through the estimated 3D RFs (schematized at left). Even with only 1 minute of training data, the ALDsf estimate recovered the qualitative structure of the RF at all time points, including the filters' departure from spacetime separability (i.e., the center pixel has different timecourse than surround). By contrast, the ML estimate is indistinguishable from noise in many places, indicating that ALDsf can reveal qualitative structure that is not visible in the ML estimate. We examined 3 ON and 3 OFF RGCs, and found that error was 18 times higher in ML estimates and 6 times higher in ridge regression estimates than in ALDsf (where error was computed with respect to the ML estimate using a full 20 minutes of data).

### Quantifying uncertainty: Bayesian confidence intervals

How can we quantify uncertainty in a receptive field estimate? The error bars shown in Figs. 5 and 6 represent variability in 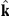 across resampled or permuted datasets. However, we would like to be able to measure the uncertainty in a single estimate given a single set of training data. Given the hyperparameters 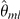, the model specifies a Gaussian posterior (eq.9) with mean 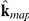 and covariance 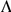. The diagonal of 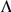 specifies the posterior variance for each element of 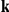, giving us 95% credible intervals (Bayesian confidence intervals) of the form
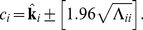
(14)The interpretation of these credible intervals is that, given the data and 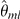, 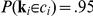. More generally, for any unit vector 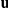, the credible interval of size (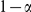) for the projection 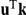 is 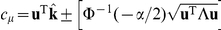, where 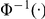 is the inverse normal cumulative density function.

However, these credible intervals, and the associated Gaussian posterior for 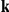, are conditioned on maximum-evidence estimate of the hyper-parameters 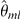. These intervals fail to take into account uncertainty in 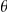, which may be substantial if the evidence 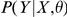 is not tightly concentrated around its maximum. The true uncertainty in 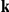 will therefore generally be greater than that captured by the posterior covariance 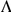.

### Fully Bayesian inference

To accurately quantify uncertainty, we may wish to perform fully Bayesian inference under the priors introduced above. Empirical Bayes (EB) inference can be interpreted as an approximate form of fully Bayesian (FB) inference in a hierarchical model [35], [45]. If we incorporate a prior 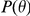 over the hyperparameters at the top level of the graphical model shown in Fig. 1 B, also known as a *hyperprior*, we will have a complete hierarchical model of the neural response. The difference between EB and FB inference for 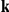 comes down to the fact that the FB prior involves marginalizing over 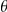:
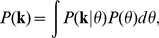
(15)while the EB prior is just the conditional distribution 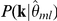. When are these priors equivalent or, more importantly, when do the EB and FB estimates agree?

The relationship between EB and FB inference can be understood by examining the posterior distribution over 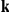. The full posterior is
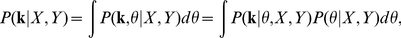
(16)where 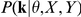 is the posterior over 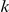 given 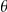, and 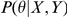 is proportional to the evidence (i.e. the exponential of (eq.10)) times the hyperprior:
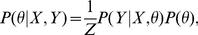
(17)where 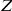 is a normalizing constant. Note that if the evidence is proportional to a delta function at its maximum, then the posterior over 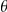 is itself a delta function, 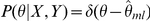. The full posterior then reduces to
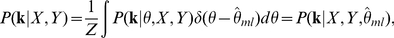
(18)which is the EB posterior (i.e., the posterior over 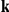 conditioned on 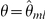). Thus, EB and FB inference are identical when the evidence is proportional to a delta function, and the two methods will in general give similar results whenever the evidence is highly concentrated around its maximum [45]. In general, EB and FB estimates will always agree given enough data, since by central limit theorem, the evidence will concentrate around its maximum with variance that falls as 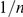. However, for finite datasets, the two may differ.

To examine the proximity of EB and FB estimates and credible intervals, we developed a sampling-based algorithm to perform FB inference under the ALD prior. The factorization shown in (eq.16) suggests an efficient method for sampling from 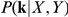 via Markov Chain Monte Carlo (MCMC), using a Markov chain over the space of the hyperparameters whose stationary distribution is proportional to the evidence. The summary of the algorithm for sampling 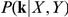 is as follows:
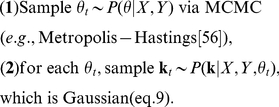
(19)A nice feature of this approach is that the hyperparameters live in relatively low-dimension (e.g., 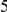 for a 1D filter and 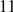 for a 2D filter under ALDsf). The Markov Chain therefore only has to explore this low-dimensional space, instead of the high-dimensional space of 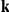, which contains tens to thousands of parameters in typical cases [57]. Samples 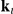 are obtained by drawing from the Gaussian conditioned on each MCMC sample 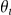. These samples may be averaged to the posterior mean 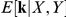, also known as the *Bayes Least-Squares* estimate, and their quantiles provide credible intervals. (See Method).

**Figure 9:**
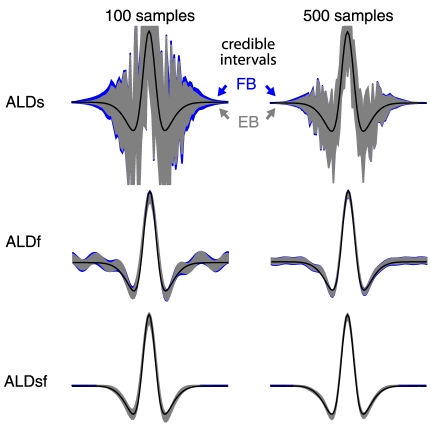
Empirical Bayes (EB) and fully Bayes (FB) credible intervals on simulated data. **Left**: FB and EB 95% credible intervals, computed from 100 samples of training data, for ALDs (above), ALDf (middle), and ALDsf (bottom). The true filter is shown in black. FB intervals are larger than EB intervals, due to the incorporation of uncertainty in the hyperparameters under fully Bayesian inference. **Right**: Credible intervals computed from 500 samples of training data. As the amount of training data increases, the FB and EB credible regions became indistinguishable, indicating that the evidence is tightly constrained around its maximum. For both amounts of training data, the posterior mean under FB and EB were virtually identical.

**Figure 10:**
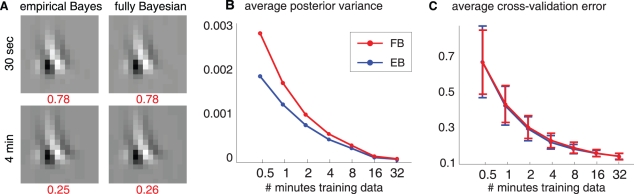
Empirical Bayes (EB) and fully Bayesian (FB) estimates on V1 data. (**A**) ALDsf estimates for a single V1 simple cell under EB and FB inference, from 30 seconds (above) and 4 minutes (below) of training data. There was no significant difference in cross-validation error (numbers below in red, averaged over 100 resampled training sets). (**B**) Marginal posterior variance of RF coefficients, averaged across pixels and across all 16 cells, under EB and FB inference. As expected, FB estimates of the posterior variance were higher, especially for small datasets, reflecting the effects of posterior uncertainty in the hyperparameters. (**C**) Average cross-validation error across 16 cells for FB and EB estimates. For all amounts of training data, error rates were nearly identical, indicating that the FB posterior mean (computed via MCMC) is not superior to the more computationally inexpensive EB estimate.

Fig. 9 shows a comparison of EB and FB estimates and credible intervals for the 1D simulated example shown previously. The hyperprior 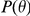 was taken to be uniform over a large region (See Methods). For a small dataset, the FB credible intervals were noticeably larger than the EB credible intervals, as expected, owing to the effects of uncertainty in 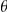 [35]. For larger datasets, this discrepancy was much smaller, and was smaller in general for ALDsf than ALDs or ALDf intervals. The EB and FB (Bayes least-squares) filter estimates, however, did not differ noticeably even for small amounts of data. Fig. 10 shows a comparison of EB and FB inference for the V1 neural data presented in Fig. 6. For small datasets, the FB credible intervals were larger than EB intervals, but cross-validation error did not differ noticeably across dataset sizes. This suggests that the higher computational cost of FB inference may not be justified unless one is interested in obtaining accurate quantification of uncertainty from a small or noisy dataset.

## Discussion

We have described a new family of priors for Bayesian receptive field estimation that seek to simultaneously exploit locality in spacetime and spatiotemporal frequency. We have shown that empirical Bayes estimates under a localized prior are more accurate than those obtained under alternative priors designed to incorporate sparsity and smoothness. Although the ALD prior does not explicitly impose sparseness or smoothness, the estimates obtained with realistic neural data were both sparse and smooth. Sparsity arises from the fact that pixels outside a central region fall to zero, while smoothness arises from the fact that Fourier coefficients outside some low-frequency region fall to zero. However, for a receptive field dominated by high frequency components, ALD should outperform ASD and other smoothed estimates (e.g., smooth RVM [47], fused lasso [58]), since it can also select regions centered on high frequencies.

We have also derived an algorithm for performing fully Bayesian inference under ALD, ASD, and ridge regression priors. The algorithm exploits the low-dimensionality of the hyperparameter space and the tractability of the evidence to perform MCMC sampling of the posterior over hyperparameters. The full prior takes the form of a *Gaussian scale mixture* [59], [60], a mixture of zero-mean Gaussians with covariances 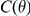 and mixing weights 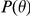, resulting in a Gaussian posterior over 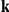 given 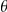 that is trivial to sample. MCMC sampling allows for the calculation of fully Bayesian credible intervals over RF coefficients, which we found to be systematically larger than empirical Bayesian intervals. Nevertheless, we found no differences in the quantitative performance of EB and FB receptive field estimates with either simulated or real neural data (Figs. 9 and 10). Of course, both intervals rely on the linear-Gaussian model of the neural response, which may be inaccurate in cases where the neural response noise is highly non-Gaussian (e.g., heavy-tailed).

More generally, this work highlights the advantages of locality as an additional source of prior information in biological inference problems. Shrinkage and sparsity have attracted considerable attention in statistics, and they have advantageous properties for a variety of high-dimensional inference problems [29], [50], [61], [62]. ALD exploits a stronger form of prior information, assuming that large groups of coefficients go to zero in a correlated manner. This may not hold for generic regression problems; for a sparse filter with randomly distributed non-zero coefficients, the ARD estimate substantially outperforms ALD (not shown), but such filters are unlikely to arise in neural systems.

Two general ideas that arise from ALD may be useful for thinking about statistical inference in other biological and non-biological systems. The first is the idea of exploiting an underlying coordinate system or topography. Whenever the regression coefficients can be arranged topographically (e.g., temporally, spatially, spectrally), it may be possible to design a prior that exploits dependencies within this topography using a small number of hyperparameters. This idea is central to ALD as well as to ASD, which uses the distances between RF pixels to set their prior correlation. But other coordinates and prior parameterizations are possible. For example, although ALD performs reasonably well for a simulated grid cell (Fig. 4 D), locality in space does not hold for grid cells, and a prior that exploits the “natural” parameters of grid cell responses (e.g., grid spacing, size, orientation, phase) might perform even better. Optimizing the hyperparameters governing such a prior is tractable with empirical Bayes. The second idea that arises from ALD is that of simultaneously constraining a set of regression coefficients in two (or more) different bases. The ALDsf method combines a local prior in a spacetime basis and a local prior in Fourier basis via a “sandwich matrix” (eq.13), which effectively applies prior constraints in series: first in spacetime and then in frequency. Another solution would be to combine the two priors symmetrically, e.g., using prior covariance 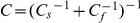. (This is the covariance that results from taking the product of the ALDs and ALDf Gaussian priors). We found this formulation to perform slightly worse on test data, but results were similar. Note that the sum of prior covariances 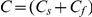 would *not* achieve the desired goal of imposing the prior constraints simultaneously, since it would prune only those coefficients in the (effective) null space of both 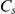 and 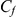. A large literature has examined regularization and feature selection in overcomplete dictionaries (e.g., “basis pursuit”) [62]–[65], but combining structured prior information defined in different bases poses an intriguing open problem.

One potential criticism of ALD is that the linear-Gaussian encoding model (eq.1) is overly simplistic. Despite its simplicity, this model has a long history in the neural characterization literature [5], [11], [18], and the estimators considered here are consistent (i.e., converge asymptotically) for responses generated by any linear-nonlinear response model, so long as the stimuli are elliptically symmetric and the expected STA is non-zero [20]. We addressed whether the linear-Gaussian modeling assumption undermines our results by re-analyzing the V1 simple cell data with maximally informative dimensions (MID) [66], an information-theoretic estimator that incorporates neural nonlinearities and Poisson spiking. The results (shown in Supporting Information (Text S1), Fig. S1), indicate that MID errors were large, comparable in size to those of the maximum likelihood (linear regression) estimate. Even when comparing to the MID filter computed from test data, ALDsf outperformed MID by a substantial margin. This shows that the limitations of the linear-Gaussian model do not substantially undermine its performance on simple cells. However, we have applied ALD only to neurons whose responses exhibit a quasi-linear relationship to the stimulus. ALD would indeed fail for a neuron with a symmetric nonlinearity (e.g., squaring) and cannot recover multiple filters (e.g., those driving a complex cell). A variety of techniques exist estimating multi-dimensional feature spaces (e.g., spike-triggered covariance (STC) [67]–[69], MID [20], [66], iSTAC [70], spike-triggered ICA [71]). However, the “kernel trick” [17], [41], which involves using linear methods on nonlinearly transformed stimuli, provides the simplest method for extending ALD to nonlinear response models. Many nonlinear transformations (e.g., transforming the stimulus to its Fourier power [72]) preserve the topography of the underlying stimulus, making this approach directly applicable to ALD.

One advantage of the linear-Gaussian model is its computational tractability. ALD is fast because the evidence can be calculated and optimized entirely from the sufficient statistics 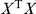, 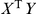, and 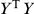 (the raw stimulus covariance, the STA, and sum of squared responses, respectively). This means that the computational cost does not scale with the amount of data (unlike MID and maximum-likelihood point process methods). Evidence optimization is also much faster than cross-validation, particularly with the 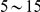 hyperparameters employed by ALDsf. The computational cost of ALD is still at least 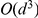 in the number of filter coefficients, since evidence evaluation requires left-division by matrices of size 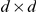. However, the number of approximately zero coefficients often falls considerably during optimization, and eliminating these coefficients by thresholding small eigenvalues of 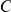 can speed convergence considerably.

Given the hyperparameters, the log-posterior over 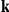 is concave, with a single maximum that can be computed in closed form (eq.5). Although the log-evidence (eq.10) is *not* concave in the hyperparameters 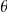, there are far fewer hyperparameters than parameters, making ALD far easier than non-convex optimization in the full space of 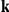 (e.g., as in MID). We can maximize the evidence more rapidly by using its first and second derivatives, which we can compute analytically (see Methods). We also exploit a heuristic strategy for initializing the ALDsf hyperparameters using the estimates from ridge regression (to identify the scale), ALDs (to identify a spatiotemporal region) and ALDf (to identify a Fourier region). Although it is substantially more computationally expensive, the fully Bayesian estimate based on MCMC avoids the issue of local maxima because it explores the entire evidence surface, not just its modes.

However, we do not ultimately view ALD and other model-based or information-based methods as in conflict. Rather, we regard ALD as providing a prior distribution over RFs that can be combined with any likelihood. Computing and optimizing the evidence under nonlinear models with non-Gaussian noise represents an important direction for future work. We suggest that locality is a general feature of neural information processing and anticipate that it will be useful for neural characterization in a wide variety of brain areas, including those where response properties are not yet well understood [73]. We expect hierarchical models and empirical and fully Bayesian inference methods to find application to a wide range of problems where structured prior information can be usefully defined.

## Methods

### Implementation of RF estimators

#### Ridge regression

For simulated and real datasets, we computed the empirical Bayes ridge regression estimate as follows. First, we initialized the noise variance to 
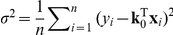
 where 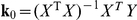 was the ML estimate, and the inverse prior variance to 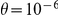. Then, we ran an iterative fixed-point algorithm [36], [45] to optimize the evidence for 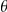 and 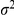,
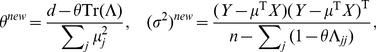
(20)where 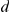 is the parameter dimensionality (number of RF coefficients), 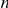 is the number of samples, 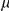 is the posterior mean (eq.9), and 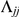 are the diagonal elements of the posterior covariance. The posterior mean and covariance are recomputed after each update to 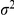 and 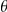. Note that (following [11], [36]) we treat the noise variance 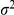 as a hyperparameter, maximizing instead of integrating it out, which is computationally more tractable, although technically it appears in the likelihood rather than the prior.

#### Automatic Relevance Determination (ARD)

We initialized the noise variance and ARD hyperparameters using the maximum-evidence values obtained from the ridge regression prior: 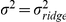 and inverse prior variance 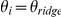. Then, we updated 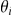 and 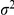 using the fixed-point rule given in [36]:
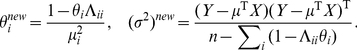
(21)


#### Lasso

We computed the Lasso estimate using the algorithm introduced in [74], [75], using software available at http://www-stat.stanford.edu/~tibs/glmnet-matlab. This implementation performs cyclical coordinate descent in a pathwise fashion. We used a test dataset with 2000 samples to find the optimal value of the lasso parameter (Fig. 2).

#### Automatic Smoothness Determination (ASD)

We computed the ASD estimate by gradient ascent of the log-evidence function, following the methods in [11]. Briefly, we initialized using the hyperparameter estimates from ridge regression: 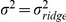, and 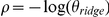, initialized the smoothness parameter 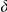 to 1, then minimized the negative log-evidence for 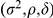 using analytically computed gradients (provided in [11]) and Hessians, which we derive below. We performed minimization using 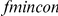 in MATLAB, with boundary conditions for the hyperparameters and the noise variance set to 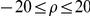, 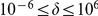, and 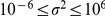, which we selected to be far larger than the range of probable values.

#### Automatic Locality Determination (ALD)

We computed ALD estimates by numerical optimization of the log-evidence using the analytically computed gradient and Hessian (second derivative matrix). For notational convenience, we will denote 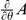, the first derivative of a quantity 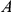 with respect to a parameter 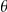, as 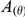, and denote the second derivative 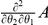 as 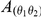.

The first derivatives of the log-evidence 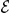 with respect to the hyperparameters 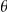 and the observation noise 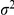 are given by [11]:
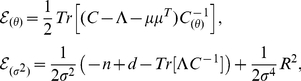
(22)where 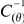 is the derivative of 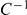 with respect to 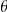, 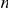 is the number of training samples, 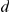 is dimensionality of 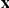, 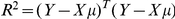 is the squared residual error, and 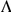 and 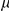 are the posterior covariance and mean, respectively (eq.9). The corresponding second derivatives are given by:
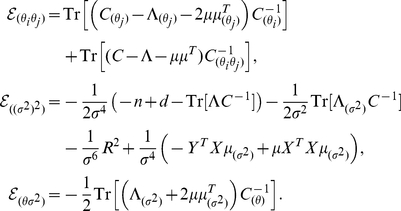
(23)These expressions involve the derivatives of 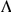 and 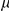 and with respect to 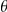 and 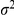, which are matrices and vectors of the same size as 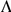 and 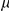, given by:
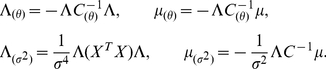
(24)


Here, 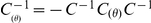. Note that 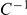 is numerically unstable when 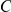 becomes ill-conditioned. Thus we never compute the inverse 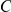 explicitly. Instead, we exploit the Woodbury matrix identity to compute the evidence and other quantities using matrices that are well-conditioned. The resulting expressions involve matrix left division instead of inversion (computed via the backslash operator in Matlab; see Supplementary Information for details). Code is available from the last authors website (http://pillowlab.cps.utexas.edu/code.html). Below, we provide the partial derivatives 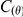 for the various ALD hyperparameters, which are all that is required for computing the gradient and Hessian of 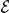.

#### ALD in spacetime (ALDs)

The hyperparameters governing the ALDs prior covariance matrix 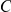 are (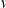, 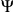, 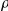), where 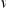 defines the mean of the localized RF, 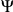 defines its elliptical extent, and 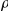 defines the scale of the prior variance (as in ASD). For filters with coordinate dimension 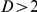, 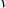 is a vector and 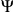 is a 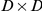 matrix that we parametrized (e.g., for 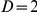) as
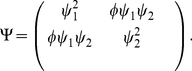
(25)For a one-dimensional filter, 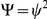, while for 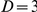, we have 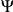 defined in terms of six hyperparameters 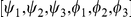. The first and second derivatives of 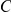 with respect to these hyperparameters (for 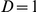) are given by:
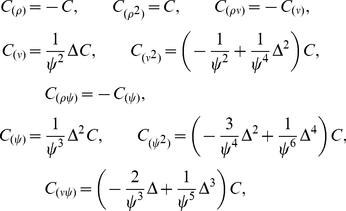
(26)where 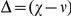 is a matrix of differences between pixel coordinates in spacetime and 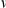.

During optimization, local maxima can be a problem if one initializes with a prior region that does not cover the location where the receptive field is largest. To avoid local maxima, we initialized 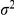 and 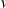 to the noise variance from ridge regression and to the center of mass of the ridge regression estimate, respectively. We used a coarse grid search for initializing 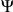, with off-diagonal terms 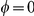. From the best initial point, we then descended the negative log-evidence using 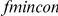 in MATLAB, with analytically computed gradients and Hessians given above. Hyperparameters were constrained to fall within the ranges 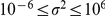, 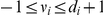, 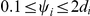, 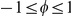, 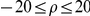, where 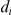 is the number of filter elements along the 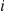
'th coordinate dimension.

#### ALD in spatiotemporal frequency (ALDf)

We implemented ALDf using the method similar to that described above for ALDs, after performing an orthogonal fast Fourier transform (FFT) on the stimuli 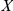, which amounts to a change-of-basis (i.e., multiplication by a unitary matrix).

As described in Results, for filters with coordinate dimension 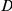, 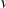 is a 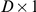 vector. 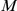 is a 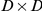 symmetric matrix, parametrized as
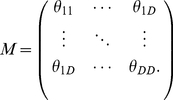
(27)


The first and second derivatives of 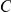 in 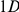 with respect to these hyperparameters are given by:
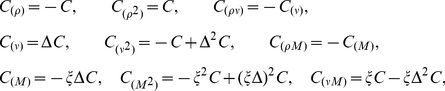
(28)where 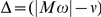 is a matrix of differences between pixel coordinates in frequency and 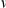, and 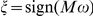.

Analogously to ALDs, to avoid local maxima, we initialized 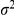 and 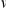 to the noise variance from ridge regression and to the centroid of the region of maximal power in the Fourier transform of the ridge-regression estimate, respectively. We used a coarse grid search for initializing 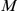. From the best initial point, we then performed optimization as described above, with boundary conditions for the noise variance and hyperparameters 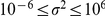, 
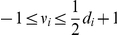
, 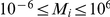, 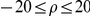, where 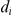 is the number of filter elements along the 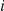
'th coordinate dimension. Once we found the filter estimate in Fourier domain, we projected it back to the spacetime domain via the inverse FFT.

#### ALD in spacetime and frequency (ALDsf)

For the jointly localized prior, we first obtained the maximum-evidence estimates for the ALDs and ALDf covariance matrices 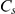 and 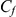 (eqs. 11 and 12). We then performed the optimization of the log-evidence for the full set of ALDsf hyperparameters using *fmincon* in MATLAB, using analytic gradient and Hessian (introduced above), with the boundary conditions for the noise variance and hyperparameters set to the same values as above.

### Application to simulated and real neural data

For the simulated data shown in Fig. 5 , we used a 2-dimensional Gabor filter (shown in Fig. 4 A) and two types of stimuli: Gaussian white noise and “naturalistic spectrum” noise–Gaussian noise with a 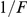 power spectrum. Simulations were carried out with various numbers of stimulus samples 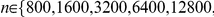 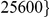, noise variance 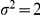, signal variance of 1, and a 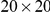 pixel filter (coordinate dimension 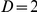, filter dimension 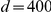). To quantify performance, we defined the filter error 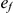 as 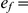 
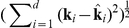
, where 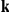 is the true filter and 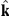 is an estimate. To obtain reliable estimates of mean error, we ran 100 simulations at each sample size. To calculate the relative error (Fig. 5 B and D), we computed the error 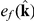 for each method, and then computed the geometric mean of the error ratio 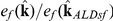 across datasets.

For V1 data shown in Fig. 6 , the data and experimental methods are described in [53]. Briefly, cells were stimulated with 1D spatiotemporal binary white noise stimuli (“flickering bars”) aligned with each neuron's preferred orientation. Stimuli were presented at a frame rate of 100 Hz. The number of bars 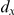 varied for different neurons, 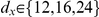. The linear receptive field was assumed to extend over a time window of 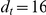 frames before a spike (a 160 ms time interval). The full dimensionality of the filter was thus 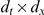, ranging from 192 to 384 parameters.

For retinal ganglion cell data shown in Fig. 8 , the data and experimental methods are described in [54], [55]. Briefly, cells were stimulated with the spatiotemporal binary white noise stimuli presented at a frame rate of 120 Hz, contained in 10×10 pixels in space. We assumed the size of the linear receptive field to be 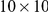 pixel 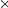
25 time bin, making for 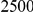 total coefficients in the RF.

We used cross-validation to quantify the performance of the various estimators (Fig. 6), and resampled the training data to examine performance as a function of training sample size. To quantify error reliably, we performed 100 repetitions for each sample size, drawing the training data randomly without replacement in blocks of size 2s, which helped to minimize the effects of non-stationarities in the data. To quantify cross-validation performance, we used relative cross-validation 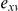, defined as 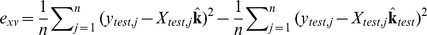, where 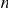 is the number of samples of test data, 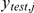 is a spike count in the 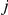
'th time bin in the test set, 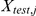 is the 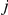
th row of the design matrix 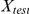, 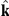 is the RF estimate obtained by each method (from training data), and 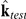 is the ML estimate obtained on the test data. Essentially, this is the ordinary test error minus the error of the ML estimator trained on test data (which provides an absolute lower bound on the performance of any linear model). We computed the relative cross-validation errors from five methods (ML, Ridge, ARD, ASD, and ALDsf) using 8 minutes of test data. In Fig. 6, we normalized the errors by dividing them by maximum average error across methods (the ML estimate using 30 seconds of data yielded the maximum cross-validation error). We computed the standard deviation of the normalized cross-validation error across 100 different training sets for each dataset size.

### Fully Bayesian inference (MCMC)

To perform fully Bayesian inference, we used Metropolis-Hastings (MH) sampling to sample from the distribution over hyperparameters 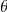 given the data 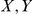. We used an isotropic Gaussian proposal distribution with variance given by the largest eigenvalue of inverse Hessian of the log-evidence around 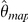. (More advanced proposal distributions and sampling methods are found in [76], [77], but this simple proposal sufficed for our purposes and mixed reasonably quickly). Thus, we first optimized the evidence to obtain the mode 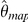 of 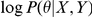, which is the mode of 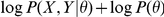. We assumed a non-informative hyperprior 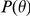, taken to be uniform over the range of values permitted during constrained optimization of the log-evidence (see above).

To carry out MH sampling, we sampled from the Gaussian proposal distribution centered on the current state 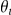 of the Markov chain, 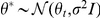, then computed 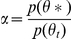, with the 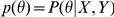. We accepted the proposal randomly with probability 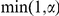, setting 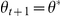, and otherwise rejected it, setting 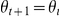. Given each sample 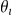, we drew a sample of the receptive field 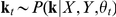. These samples were averaged to compute the posterior mean (or Bayes Least Squares estimator). Their quantiles were used to compute credible intervals for each filter coefficient.

In Fig. 10 , we compared fully Bayesian (FB) and empirical Bayes (EB) filter estimates obtained from V1 simple cell data [53]. For each set of training data, we drew 5000 samples using MH to compute the posterior mean and credible intervals. The average acceptance rate of the MH sampler was 0.12. For Fig. 10 A, we computed the average of the EB and FB error from 100 repetitions with independently drawn sets of training data. We computed the average cross-validation error of both estimates of the example cell (in red). For Fig. 10 B, we computed the average posterior variance by averaging the posterior variances in the estimates from the 100 iterations in each cell, which we then averaged across all 16 cells. For Fig. 10 C, we computed the average cross-validation error by averaging the errors from the 100 iterations in each cell, and we averaged these across 16 cells. The same 8 minutes of held out test data was used for cross-validation, for all training iterations.

## Supporting Information

Figure S1 Figure S1 shows the comparison of ALD and MID estimates.
(EPS)

Text S1 Supporting information to 1) compare the performance of the ALDsf estimator under the linear Gaussian model to the MID estimator which is equivalent to the maximum likelihood estimator under the linear-nonlinear Poisson cascade model; 2) provide expressions for the quantities for computing the log-evidence.
(PDF)
