# Supplementary material for: Receptive Field Inference with Localized Priors
Source: PLoS Comput Biol. 2011 Oct 27;7(10):e1002219. doi: 10.1371/journal.pcbi.1002219 (PMC3203052; doi:10.1371/journal.pcbi.1002219)
Supplement: Text S1 — Supporting information to 1) compare the performance of the ALDsf estimator under the linear Gaussian model to the MID estimator which is equivalent to the maximum likelihood estimator under the linear-nonlinear Poisson cascade model; 2) provide expressions for the quantities for computing the log-evidence. (PDF) [file pcbi.1002219.s002.pdf]

# Supporting Information

## 1 Comparison to MID estimator

One reasonable concern is that our results might reflect limitations of the linear Gaussian encoding model rather than the virtues of the ALD prior. That is, the evidence optimization framework relies on a linear Gaussian model of the neural response (Fig. 1), which fails to take into account neural response nonlinearities or the discrete noise distribution underlying spike counts; perhaps the maximum likelihood estimator under a more realistic encoding model would perform better than any of the estimators considered here, making ALD unnecessary.

To address this possibility concretely, we compared the performance of ALDsf to the MID (maximally informative dimensions) estimator [1], which is equivalent to the maximum likelihood estimator under the linear-nonlinear Poisson cascade model [2, 3]. This estimator takes the neural nonlinearity into account, and models the response noise as Poisson, which is clearly more accurate than Gaussian. We fitted the MID estimate using a spline with 6 knots to parametrize the distributions $P(x)$ and $P(x|spike)$, which are the necessary ingredients for computing the "single-spike information" (or equivalently, log-likelihood).

We fit MID, ALD, and Gaussian ML estimates to the data of the same V1 cell shown in (Fig. 6 left) for 100 different resamplings of the original data, for each size dataset. We used the MID estimate computed on 25 minutes of independent data as our "test" filter estimate, and computed the mean squared error between each "training estimate" and this test filter (If anything, this comparison should favor MID, since the comparison filter was computed using the same model).

Figure S1 shows that MID error rate was comparable to the Gaussian-ML estimate (i.e., linear regression), and that ALDsf achieved significantly lower error. This demonstrates that benefits conferred by the ALD prior are not compromised by the assumption of a linear Gaussian response model. In fact, the MID estimate performed slightly worse than the linear regression estimate, perhaps due to the fact that it effectively has more free parameters (including those governing the nonlinearity) and therefore has even greater need of regularization.

## 2 Essential quantities

Here we provide expressions for many of the quantities required for computing the log-evidence $\mathcal{E}$, which are useful for numerical optimization of the ALD model parameters. Although these expressions are all available in the published literature [4–6], it is useful to have them compiled in one place, using the same notation.

More importantly, we provide expressions for evaluating the evidence and posterior mean that avoid inverting the prior covariance $C$ or the posterior covariance $\Lambda$. This is important for cases where the prior becomes ill-conditioned due to pixels or frequencies are effectively pruned from the model. We use the $\backslash$ ("backslash" operator in matlab) to indicate left-division, which is a faster and more numerically stable way to left-multiply by the inverse of a matrix. (Note that the matrices to which we apply the backslash operator here are always well-conditioned).

**Likelihood.** from linear-Gaussian encoding model:

$$P(Y|X,\mathbf{k},\sigma^2) = \frac{1}{|2\pi\sigma^2 I|^{\frac{1}{2}}} \exp\left[-\frac{1}{2}(\mathbf{k}-m)^\top L^{-1}(\mathbf{k}-m)\right]$$
$$= |L|^{\frac{1}{2}}\sigma^{-n}\mathcal{N}(m,L) \tag{1}$$

where

$$L = \sigma^2(X^TX)^{-1} \quad \text{and} \quad m = \frac{1}{\sigma^2}LX^\top Y = (X^TX)^{-1}X^\top Y. \tag{2}$$

Note $L^{-1} = \frac{X^TX}{\sigma^2}$.

**Prior.** Zero mean Gaussian with covariance $C$:

$$P(\mathbf{k}|\theta) = \mathcal{N}(0, C(\theta)). \tag{3}$$

**Posterior.** Gaussian, proportional to product of likelihood and prior:

$$P(\mathbf{k}|X,Y,\theta,\sigma^2) = \mathcal{N}(\mu,\Lambda) \tag{4}$$

where

$$\Lambda = (L^{-1}+C^{-1})^{-1} \tag{5}$$
$$= (\tfrac{1}{\sigma^2}CX^TX + I_d)^{-1}C \tag{6}$$
$$= \left(\tfrac{1}{\sigma^2}CX^TX + I_d\right)\setminus C, \tag{7}$$

and

$$\mu = \Lambda L^{-1}m = \tfrac{1}{\sigma^2}\Lambda X^\top Y \tag{8}$$
$$= (X^TX + \sigma^2 C^{-1})^{-1}X^\top Y \tag{9}$$
$$= \left[(CX^TX + \sigma^2 I_d)\setminus C\right]X^\top Y, \tag{10}$$

where $I_d$ is a $(d\times d)$ identity matrix and $d$ is the parameter dimensionality of $\mathbf{k}$.

**Evidence:**

$$P(Y|X,\theta,\sigma^2) = \int P(Y|X,\mathbf{k},\sigma^2)P(\mathbf{k}|\theta)d\mathbf{k}$$
$$= \frac{|2\pi\Lambda|^{\frac{1}{2}}}{|2\pi\sigma^2 I_n|^{\frac{1}{2}}|2\pi C|^{\frac{1}{2}}}\exp\left[\frac{1}{2}\left(\mu^\top\Lambda^{-1}\mu - m^\top L^{-1}m\right)\right] \tag{11}$$

where $I_n$ is a $(n\times n)$ identity matrix and $n$ is the number of data points.

**Log-Evidence:**

Define $\mathcal{E} = \log P(Y|X,\theta,\sigma^2)$ and we have

$$\mathcal{E} = -\tfrac{n}{2}\log|2\pi\sigma^2| - \tfrac{1}{2}\log|C\Lambda^{-1}| + \tfrac{1}{2}\mu^T\Lambda^{-1}\mu - \tfrac{1}{2\sigma^2}Y^\top Y. \tag{12}$$

Special case: what happens when C goes to all-zeros (or equivalently, all coefficients are pruned)? If $C=0$, then $C^{-1}=\infty$. So $\Lambda = 0$. In this case, the log-evidence reduces to:

$$\mathcal{E} = -\tfrac{n}{2}\log|2\pi\sigma^2| - \tfrac{1}{2\sigma^2}Y^\top Y. \tag{13}$$

# References

1. Sharpee T, Rust NC, Bialek W (2004) Analyzing neural responses to natural signals: maximally informative dimensions. Neural Comput 16: 223–250.

2. Kouh M, Sharpee TO (2009) Estimating linear-nonlinear models using Renyi divergences. Network 20: 49–68.

3. Williamson RS, Sahani M, Pillow JW (2011) On information-theoretic and likelihood-based methods for spike-triggered neural characterization. In: Computational and Systems Neuroscience (CoSyNe) Abstracts.

4. MacKay D (1991) Bayesian interpolation. Neural Comput 4: 415–447.

5. Tipping M (2001) Sparse Bayesian learning and the relevance vector machine. J Mach Learn Res 1: 211–244.

6. Sahani M, Linden J (2003) Evidence optimization techniques for estimating stimulus-response functions. Advances in Neural Information Processing Systems 15: 301–308.

7. Rust NC, Schwartz O, Movshon JA, Simoncelli EP (2005) Spatiotemporal elements of macaque V1 receptive fields. Neuron 46: 945–956.

**Figure S1. Comparison of MID and ALDsf estimates.**
**Left**: RF estimates from MID, ML (linear Gaussian model), and ALDsf, using one minute of data from a V1 simple cell (data from [7]). **Right**: Mean squared error between MID, ML and ALDsf estimates and an MID estimate computed on an independent 25m test set, as a function of the amount of training data. Each point represents an average of 100 training datasets randomly sub-sampled from the original data.
